# Recombination-ready Sindbis replicon expression vectors for transgene expression

**DOI:** 10.1186/1743-422X-4-112

**Published:** 2007-10-26

**Authors:** Brian J Geiss, Lisa H Shimonkevitz, Cherilyn I Sackal, Ken E Olson

**Affiliations:** 1 Arthropod-Borne and Infectious Diseases Laboratory, Department of Molecular Biology. Immunology, and Pathology, Colorado State University, Fort Collins, CO 80523 USA

## Abstract

**Background:**

Sindbis viruses have been widely used as tools to study gene function in cells. Despite the utility of these systems, the construction and production of alphavirus replicons is time consuming and inefficient due to potential additional restriction sites within the insert region and lack of directionality for insert ligation. In this report, we present a system useful for producing recombinant Sindbis replicons that uses lambda phage recombination technology to rapidly and specifically construct replicon expression plasmids that contain insert regions in the desired orientation.

**Results:**

Recombination of the gene of interest with the replicon plasmid resulted in nearly 100% recombinants, each of which contained a correctly orientated insert. Replicons were easily produced in cell culture and packaged into pseudo-infectious viral particles. Insect and mammalian cells infected with pseudo-infectious viral particles expressed various transgenes at high levels. Finally, inserts from persistently replicating replicon RNA were easily isolated and recombined back into entry plasmids for sequencing and subsequent analysis.

**Conclusion:**

Replication-ready replicon expression plasmids make the use of alphavirus replicons fast and easy as compared to traditional replicon production methods. This system represents a significant step forward in the utility and ease of use of alphavirus replicons in the study of gene function.

## Background

Alphaviruses, such as Sindbis virus (SINV), are positive-sense RNA viruses that have been extensively used for transgene expression in mammalian and insect systems. SINV are ideal for transgene expression because they can express high levels of exogenous protein or RNA [1,2], infect a wide range of species [1,3], stably replicate for long periods of time with minimal cytotoxicity [1,3,4], and do not integrate into the host genome. Two main approaches have been used for SINV transduction systems: 1) non-infectious subgenomic replicons that express exogenous genes from the native subgenomic promoter (SGP) that normally would express the structural proteins, and 2) infectious SINV containing an engineered second SGP for gene expression. Subgenomic replicons are capable of authentic viral RNA replication and can express exogenous genes, but are unable to form viral particles capable of intercellular spread because they lack structural proteins that form virus particles. The lack of intercellular spread by the replicons allows for the isolation of clonal cell populations that express a gene of interest, which is useful if the replicon is being used to generate a homogenous population of cells. SINV replicons have been used in a wide range of applications, including expression of reporter genes [5], gene therapy [6], vaccination [7], and expression of heterologous viral proteins [8]. The addition of a drug resistance gene to a replicon, such as blasticidin S-deaminase (bsd) or puromycin N-acetyltransferase [5], can result in long term persistent infection and selection for cells containing replicons. Because the SGP is small and well defined, SINV replicons containing multiple SGPs have been generated that allow for simultaneous drug selection and transgene expression [5]. Infectious SINV containing a second SGP have recently been used to express green fluorescent protein *in vivo*, allowing real-time visualization of viral infection in mosquito vectors [9,10]. Co-transfecting replicon containing cells with a plasmid that expresses the viral structural proteins can produce pseudo-infectious viral particles (PIPs) that can initiate a single round of infection but are unable to spread to additional cells. PIPs are useful because they can be generated at high titers and have the same tropism as infectious SINV but are not able to spread from the cell they infect. Thus SINV replicons are a valuable tool for transgene expression both in cell culture and in live animals.

While SINV replicons are very useful for studying gene expression, they historically have had several drawbacks that make them difficult to use. One major problem is generating and delivering replication competent replicon RNA into target cells. Replicon RNA has classically been generated by *in vitro *transcription in the presence of nucleotide cap analog, and the resulting RNA is electroporated into the cells. Transcription and RNA electroporation require specialized protocols and equipment that are not readily available in all laboratories. Replicon expression plasmids that use mammalian promoters to transcribe replicon RNA from transfected plasmids have been developed to circumvent these issues [11]. Expression plasmids allow replicons to be generated simply by transfecting plasmid DNA into mammalian cells, and the replicon RNA can be packaged into PIPs by co-transfection with a separate packaging plasmid or transfection into a packaging cell line [12].

A second issue is engineering the insert of interest into the replicon plasmid in the proper orientation. DNA coding for genes is ligated into the replicon plasmid using a unique restriction site 3' to the SGP, allowing virus-mediated transcription of the insert DNA to occur [13]. The insert DNA can be ligated into the replicon plasmid in either a sense or antisense orientation, resulting in a mixed population of recombinants that must be screened for clones of the appropriate orientation. Screening individual clones for orientation is not a difficult task if only one clone is desired, but the inability to guide insert orientation or select for recombinant replicon plasmids makes the development of replicon libraries difficult because inserts will be present in both orientations or in multiple copies. Additionally, if the restriction site used is found within the insert DNA sequence, alternate cloning strategies must be employed to avoid truncating the insert. If a library is being constructed, the complexity of the usable library will likely be reduced because many cDNAs will contain the restriction site. To address the problem of cloning inserts into SINV replicons efficiently and in the correct orientation, we have constructed a replicon expression plasmid that contain lambda phage attR Gateway™ recombination sequences 3' of the second SGP. The attR recombination sequences flank a negative selection marker that increases the rate of correct recombination and reduces the number of non-recombinant clones to be screened. The attR1 and attR2 recombination sites, which allow specific recombination between attL1 and attL2 sites in the presence of LR Clonase enzyme mixture, are positioned in either a sense or antisense orientation, allowing highly specific and directional recombination between the replicon plasmids and attL1/attL2 flanked insert sequences. The experiments described show that addition of recombination technology increases the speed and efficiency of replicon plasmid construction, make the manipulation of alphavirus replicons easier and more user-friendly to the non-expert user, and provides a foundation for the establishment of SINV replicon-mediated expression cloning protocols.

## Materials and methods

### Construction of replicon expression plasmids, donor plasmids, and packaging plasmid

We used the double-subgenomic infectious clone of the AR339 Sindbis strain pTE3'2J [14] to construct the replicons. All oligonucleotide primers used in this report are listed in Table 1. The base replicon expression plasmid, pBG68, was constructed in multiple stages. All PCR amplification was performed with Platinum Taq Hi Fidelity (Invitrogen). The cytomegalovirus immediate early (CMV) promoter from pcDNA 3.1 Zeo (+) (Invitrogen) was PCR amplified with primers BG31 and BG34 to generate Fragment A. BG34 contains a 36 nucleotide (NT) segment of the SINV 5' UTR and positions the CMV transcription start site at NT 1 of the SINV 5' UTR. A 2336 bp fragment from the start of the 5' UTR to the BglII restriction site (nucleotide 2293) was PCR amplified from pTE3'2J with primers BG32 and BG33 (Fragment B). BG32 is complementary to BG34 and adds a 14 NT of the 3' end of the CMV promoter to the PCR product to correctly position the transcription initiation site with the 5' nucleotide of the Sindbis 5' UTR. Fragments A and B were linked by overlap extension PCR[15] with primers BG31 and BG32, resulting in Fragment C, which was digested with MluI and BglII. Fragment D contains SINV sequences NT 2289 to 5263, and was PCR amplified with primers BG35 and BG36 and digested with BglII and SpeI. Fragment E contains the carboxy terminus of nsP4 and the 5' SGP from pTE3'2J (SIN NT 5264 to 7649, and was PCR amplified with primers BG37 and BG39. Fragment G contains the blasticidin-s-deaminase gene and was generated with primers BG38 and BG41 and plasmid pIB-VH (Invitrogen). Primers BG39 and BG38 contain complementary sequences and were used to link Fragments E and G, forming fragment F. Fragment H contains the second SGP, the unique XbaI restriction site, the SINV 3' UTR, and a hepatitis D virus (HDV) ribozyme [16] positioned to cleave at the SINV 3' terminal RNA nucleotide. Fragment H was initially PCR amplified from TE3'2J with primers BG40 and BG42, and the remainder of the HDV ribozyme was added to Fragment H by sequential amplification with primers BG40/BG43 (Fragment H_i_) and BG40/BG44 (Fragment H_ii_). The ribozyme containing Fragment H_ii _was linked to Fragment F by overlap extension PCR, forming Fragment I. To generate plasmid pBG53, pcDNA 3.1 Zeo (+) was digested with MluI and XhoI, and the pcDNA 3.1 vector backbone was ligated to Fragments C (MluI/BglII), D (BglII/SpeI), and H_ii _(SpeI/XhoI). An extra XbaI site remaining in the multiple cloning site of pBG53 was removed by Quickchange Mutagenesis (Stratagene), resulting in the replicon expression plasmid pBG68 that contains a unique XbaI restriction site 3' to the second SGP. pBG68 and all remaining plasmids in this report were verified by sequencing.

**Table 1:** 

Oligo	Sequence
BG31	5' ATATACGCGTTGACATTGATTATTGACTAG
BG32	5' GGTAACAAGATCTCGTGCCG
BG33	5' GGGAGGTCTATATAAGCAGAGCTCGTTTAGTGAACCGATTGACGG CGTAGTACACACTATTGAATCAAACAGCCG
BG34	5' CGGCTGTTTGATTCAATAGTGTGTACTACGCC GTCAATCGGTTCACTAAACGAGCTCTGCTTATATAGACCTCCC
BG35	5' GGCACGAGATCTTGTTACCAGCGG
BG36	5' TGTCCATACTAGTAATAGAGTTGTCC
BG37	5' GGACAACTCTATTACTAGTATGGACAG
BG38	5' CTGACTAATACTACAACACCACCACCATGGCCAAGCCTTTGTCTCAAG
BG39	5' TTGAGACAAAGGCTTGGCCATGGTGGTGGTGTTGTAGTATTAGTCAG
BG40	5' CCCTCTGGTTATGTGTGGGAGGGCTAACGGGCCCAGGTAGACAATATTACACC
BG41	5' GGTGTAATATTGTCTACCTGGGCCCGTTAGCCCTCCCACACATAACCAGAGGG
BG42	5' GGCGCCAGCGAGGAGGCTGGGACCATGCCGGCCTTTTTTTTT TTTTTTTTTTTTTTTTTTTTTTTTTTTTGAAATGTTAAAAACAAAATTTTG
BG43	5' ATTACCGAGGGGACGGTCCCCTCGGAATGTTGCCCAGCCG GCGCCAGCGAGGAGGCTGGGACCATGCCGGCC
BG44	5' GCCCGCTAGCCTCGAGGAATTCCCGTCCCATTCGCCATTACCGAGGGGACGGTCCCC
BG115	5' CATGAAGCTTATGAATAGAGGATTCTTTAACATGCTCG
BG116	5' CTACCTGGGCCCGTCATCTTCGTGTGCTAGTCAGC
BG121	5' CATGGCTAGCACAAGTTTGTACAAAAAAGCTGAACG
BG122	5' CATGGCTAGCACCACTTTGTACAAGAAAGCTGAACG
BG155	5' GGGGACAAGTTTGTACAAAAAAGCAGGCTTCGAAGGAGATAGAACCATGGGGATGCA TGGTACCATGGTGAGCAAGG
BG156	5' GGGGACCACTTTGTACAAGAAAGCTGGGTTTACTTGTACAGCTCGTCCATGCCG
BG162	5' TTGGGGCGTAGCGTCTAGGATCCATG
BG256	5' GGGGACAAGTTTGTACAAAAAAGCAGGCTTCGAAGGAGATAGAACCATGGCCTCAAAG CCAGTCCTGAGCACG
BG258	5' GGGGACCACTTTGTACAAGAAAGCTGGGTCTAATGGTGATGGTGATGATGACCG
BG192	5' TCTGGTTATGTGTGGGAGGGC
BG209	5' GGCCAAGATCTTTTCTTGCTGGTTCTCTTGTACAGCTCGTCCATGCCG

**Figure 1:**
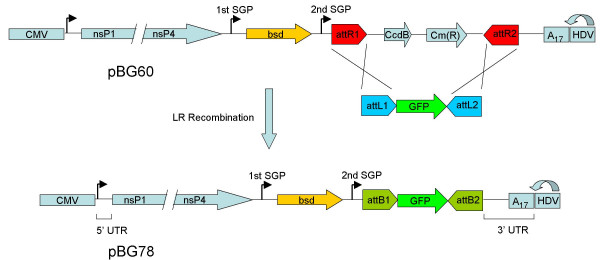
**Structure of the Gateway SINV replicon plasmids pBG60 and pBG78**. Transcription of the viral RNA is initiated by the cytomegalovirus immediate early (CMV) promoter and terminated by a polyadenylation signal 3' of the HDV ribozyme (not shown). The hepatitis delta virus (HDV) ribozyme trans-cleaves the RNA, resulting in an authentic 3' RNA end. bsd represents the blasticidin S-deaminase gene. The Gateway attR1/attR2 recombination sites are positioned 3' of the 2nd SGP. CcdB represents *E. coli *DNA Gyrase poison. Cm(R) represents chloramphenicol acetyltransferase. Recombination of the pBG60 plasmid with pBG76 results in the pBG78 plasmid.

The attR1/attR2 recombination cassette was PCR amplified from Gateway pDEST32 (Invitrogen) with primers BG121 and BG122. BG121 and BG122 contain NheI restriction sites, and were used to ligate the recombination cassette into XbaI digested pBG68. pBG60 contains the recombination cassette in the attR1/attR2 orientation with respect to the second SGP. pBG60 was propagated in the *E. coli *strain DB3.1, which is resistant to the CcdB gene present in the attR recombination cassette. pBG60 (Figure 1) is considered a destination plasmid competent for recombination with attL1/attL2 flanked DNA sequences.

Donor plasmid pBG76 contains the green fluorescent protein gene flanked by attL1 and attL2 recombination sites. To construct pBG76, primers BG155 and BG156, which contain attB1 and attB2 recombination sites were used to PCR amplify eGFP from pIE-GFP (Clontech). The PCR product was incubated in a BP Clonase recombination reaction with entry plasmid pDONR222 (Invitrogen) for 1 hr at 25°C. The reaction was electroporated into XL-1 Blue *E. coli *cells and plated onto kanamycin-containing agar plates. Donor plasmid pBG151, which encodes a V5 epitope-tagged *Ae. aegypti *R2D2 cDNA flanked by attL1/attL2 sites, was constructed in a similar manner to pBG76 using primers BG256/BG258.

pBG44, which expresses the SINV structural proteins from a CMV promoter, was constructed by PCR amplifying the structural protein open reading frame from pTE/3'2J with primers BG115 and BG116. The PCR product was digested with HindIII and ApaI, gel purified, and ligated into the pcDNA 3.1 Zeo (+) using the same restriction sites.

All *in silico *DNA manipulation was performed with Vector NTI 10 Suite (Invitrogen).

### Recombination ready C6/36 cDNA library preparation and recombination reactions

cDNA libraries were constructed using the CloneMiner cDNA Construction Kit (Invitrogen) as recommended by the manufacturer. A representative cDNA library was prepared from actively growing C6/36 (*Aedes albopictus*) cells. Briefly, mRNA was isolated from 1.0 × 10^7 ^C6/36 cells using the Micro-FastTrack 2.0 mRNA Isolation kit (Invitrogen). cDNAs were synthesized with attB1 recombination sites at the 5' terminus of the cDNA and attB2 recombination sites at the 3' poly-A tail of the cDNA. Isolated cDNAs were recombined using BP Clonase into the pDONR222 plasmid, electroporated into DH10B *E. coli *cells, and aliquots stored as glycerol stocks at -80°C. The size of the library was determined in colony forming units/ml of glycerol stock from an individual aliquot. Multiple clones were sequenced to verify appropriate recombination. A portion of the cDNA library plasmid containing larger inserts was isolated by agarose gel extraction.

A completely defined micro-scale cDNA library containing inserts of greater that 1 Kb was generated from gel extracted plasmids derived from the C6/36 cDNA library described above. The gel-extracted plasmids were transformed into DH5α cells. Ten individual clones were isolated and the size of the insert regions was determined by BsrGI restriction enzyme digestion. The micro-scale cDNA library was generated by mixing equal molar amounts of each of the ten clones, and the micro-scale cDNA library was used in a LR Clonase reaction with pBG60. The product of the recombination reaction was transformed into DH5α cells (CcdB sensitive) and analyzed for insert size by colony PCR and BsrGI restriction digestion.

### Cell culture, transfection, and replicon packaging

Baby hamster kidney (BHK) cells were cultured in Dulbecco's modified Eagle medium supplemented with 10% fetal bovine serum (FBS) as previously described [17]. BHK cells were transfected using Lipofectamine 2000 following the manufacturer's instructions. C6/36 cells were cultured in L-15 insect medium supplemented with 10% FBS as previously described [18]. S2 cells were cultured is Schneider's media containing 10% FBS. Replicon-containing C6/36 cells were maintained in the presence of 6 ug/ml blasticidin (Invitrogen) for at least 2 weeks prior to assay, and replicon-containing S2 cells were maintained in the presence of 10 ug/ml blasticidin for 4 days prior to assay. C6/36 and S2 cells were transfected using Insect Gene Juice transfection reagent (EMD Biosciences), using a ratio of 10 μl reagent/μg DNA.

To generate PIPs, replicon expression plasmids were co-transfected with the packaging plasmid pBG44 into subconfluent BHK cells. Cells were washed extensively 4 hours after transfection to remove remaining plasmid DNA and transfection reagent. Twenty-four hours later media from the transfected BHK cells was collected, centrifuged at 13,000 × G to remove any cell debris, and the clarified supernatant was collected and frozen at -80°C. To infect C6/36 cells, the supernatant was added directly to subconfluent C6/36 cells. Twenty-four hours after infection, the media was replaced with fresh complete L-15 media containing 10 μg/ml blasticidin and the cells were selected for an additional 2 weeks. Conditions for co-culture assays are described in the results section.

GFP expression in live BHK and C6/36 cells was visualized on an Olympus inverted fluorescence microscope and images were captured with a coupled CCD camera.

### Western blot analysis

To prepare cell samples, equal numbers of C6/36 cells were boiled in 1× SDS loading buffer and clarified by centrifugation at 13,000 × G. Equal volumes were loaded and resolved on 12% SDS-PAGE gels, and proteins were transferred to nitrocellulose membranes. Membranes were incubated Anti-V5 antibody (Invitrogen), then incubated with horse radish peroxidase-conjugated anti-mouse secondary antibody. Proteins were detected with ECL Plus Western Blot Detection kit (Amersham) on a Storm 860 phosphorimager.

### Colony PCR and reverse transcription of replicon insert regions

Colony PCR was used to determine the size of inserts recombined into the pBG60 plasmid. Individual colonies from transformed BP or LR reactions were touched with a 10 μl pipette tip and mixed into a 20 μl PCR reaction containing 1 pM primers, recombinant Taq (Invitrogen), 0.2 mM dNTP, and 5% DMSO. 1 μl of the bacteria-containing colony PCR reaction was spotted onto ampicillin-containing agar plates prior to thermocycling for archiving. Colony PCR reactions were subjected to 40 cycles of (95°C (30 s)/55°C (30 s)/72°C (1 m/kb)) and products were resolved on 1% agarose gels.

RNA from replicon-containing C6/36 cells was isolated using RNeasy RNA isolation kits (Qiagen). One microgram of total RNA was reverse transcribed with replicon-specific primers BG162/BG192 using the Superscript One-Step RT PCR system (Invitrogen). The resulting PCR product was purified using a Qiaquick PCR prep kit (Qiagen), and the PCR product was recombined into pDONR222 in BP clonase reactions. Recombination reactions were transformed into DH5α E. coli cells and grown on 50 mg/ml kanamycin plates. Individual colonies were tested for inserts by colony PCR, and select clones were verified by sequencing.

## Results

### Replicon insert recombination is efficient and directional

**Figure 2:**
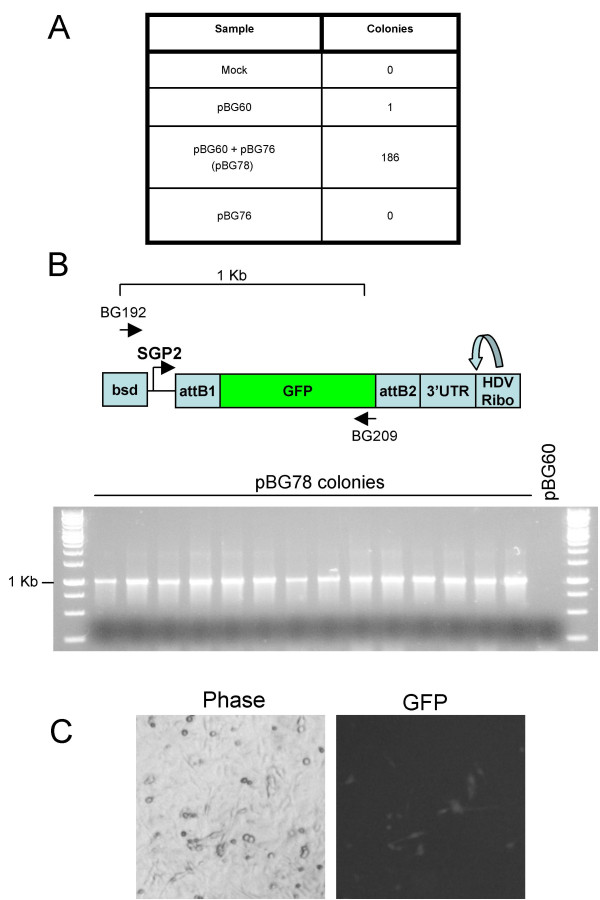
**Efficiency of recombining genes into pBG60 vector**. **A**. Bacterial colonies obtained from recombination reactions. **B**. Structure of the pBG78 2nd SGP and position of asymmetric colony PCR primer pair. **C**. Colony PCR of 14 independent clones from colonies in Figure 2A. **D**. pBG78 plasmids produce replication-competent RNA in BHK cells. BHK cells were transfected with one random pBG78 clone and fluorescence signal was detected 48 hours post transfection.

We first tested the ability of the replicon plasmid pBG60 (Figure 1) to incorporate an attL flanked insert in a LR recombination reaction. Recombination of the attR-containing replicon plasmid (pBG60) with an attL flanked GFP entry plasmid (pBG76) in a LR Clonase II reaction resulted in a large number of colonies when transformed into DH5α cells (Figure 2A), whereas transformation with pBG76 or pBG60 incubated with LR Clonase resulted in almost no colonies. Fourteen colonies from the pBG60–pBG76 recombination (referred to as pBG78, Figure 1) were examined for insert size and orientation by asymmetric colony PCR with primers BG192/BG209 (Figure 2B). All of the colonies produced a PCR product of 1 Kb, indicating that the GFP gene was present in the replicon plasmid in the correct orientation. Sequencing the insert region of several of the plasmids verified the presence and orientation of the insert, and the reconstitution of the attB1 and attB2 sites flanking the GFP open reading frame. A related replicon plasmid which contains the attR recombination cassette in the attR2/attR1 orientation with respect to the second SGP (pBG59), was similarly able to incorporate attL flanked inserts, but in reverse orientation (data not shown). Finally, transfection of the one of the pBG78 plasmid clones into BHK cells results in GFP expression 2 days after transfection (Figure 2C). Because the expression of genes downstream of the 3' SGP are dependent on replicon RNA replication, the expression of GFP indicates that the replicon is actively replicating and that the GFP gene is being expressed in a replicon dependent manner. Therefore, the presence of the attB recombination sites does not interfere with expression of the inserted gene from the SGP or replication of the replicon RNA.

### attL-flanked cDNAs of varying sizes can be efficiently recombined into replicon plasmids

**Figure 3:**
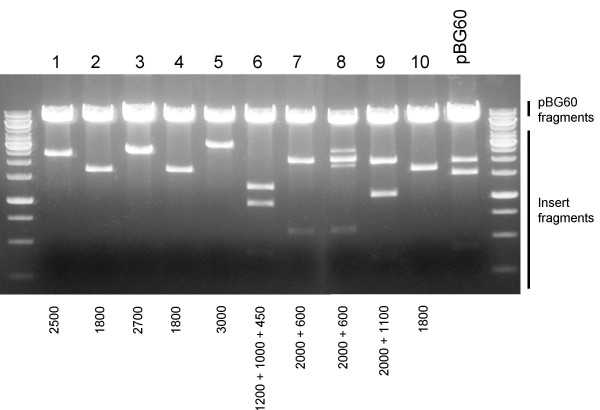
**Restriction analysis of micro-scale library clones**. Ten individual cDNA clones were recombined with pBG60 and transformed into XL1-Blue *E. coli*. Plasmid DNA was prepared from ten individual colonies picked at random and restriction digested with BsrGI determine the size of the recombined insert.

We next wanted to determine if a mixed population of attL flanked cDNAs could be recombined into the replicon plasmids and determine if large inserts (≤ 3 Kb) can be tolerated by the plasmid and replicon. We generated a representative attL-flanked cDNA entry library from C6/36 cells using the Cloneminer cDNA construction kit. The initial non-size selected cDNA library contained a large proportion of small attL-flanked inserts, so to determine how large of inserts the replicon plasmids can tolerate from a mixed population we size selected the cDNA entry plasmids for individual clones with inserts of greater that 1 Kb by gel extraction. We combined 10 individual clones of greater that 1 Kb to generate a defined micro-scale cDNA library. The micro-scale cDNA library was then used in a LR Clonase II reaction with pBG60. Ten random clones were selected from the pBG60 – micro-scale library recombination reaction and analyzed by BsrGI digestion to determine the size of the cDNA insert in each clone (Figure 3). Each of the 10 individual entry plasmid inserts produced different BsrGI digestion patterns, and we observed seven of the ten characteristic patterns in the ten pBG60-microscale library clones we tested. We observed three of the clones containing the smallest clone (Figure 3, lanes 2, 4, and 10), which may indicate that the recombination occurred somewhat more efficiently with the smaller insert than with the longer inserts. However, the presence of so many of the different inserts in our sampling indicates that complex mixtures of replicon plasmids containing different cDNA clones can be achieved. Additionally, the largest cDNA that was recombined was over 3 Kb in size (Figure 3, lane 9), demonstrating that the replicon expression plasmid can tolerate inserts of at least 3 Kb.

### Replicons can be packaged into PIPs and used to infect naive mammalian or insect cells

**Figure 4:**
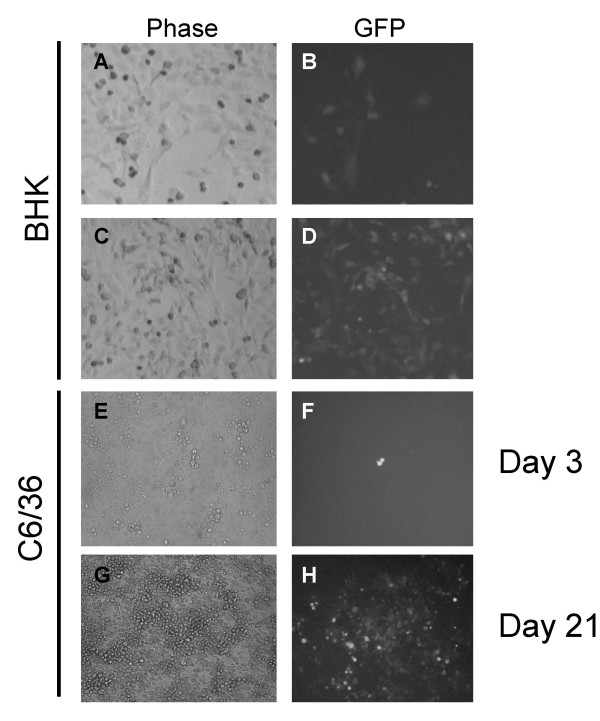
**Packaging and infection of pBG78 replicons in BHK and C6/36 cells**. pBG78 PIPS were generated by co-transfecting pBG78 and pBG44 plasmid DNA into BHK cells. Media was collected 24 hrs post transfection and 100 ul of the clarified media was added to new BHK or C6/36 cells. 24 hrs after infection the media was replaced with fresh media containing10 ug/ml blasticidin and the cultures were maintained for an additional three weeks. Media was replaced every 3–4 days. GFP fluorescence was detected at 3 days post infection (panels B and E) and 3 weeks post infection (panels D and H).

The ability of the replicon RNA to be packaged into PIPs and infect naive cells was tested. BHK cells were transfected with GFP expressing replicon plasmid pBG78 either alone or with the packaging plasmid pBG44. Transfection of pBG78 alone resulted in ~20% of the cells expressing GFP and the remaining cells were GFP negative (Figure 4, Panel b). However, co-transfection of pBG78 and pBG44 resulted in almost all the cells in the cultures expressing GFP either at high or medium levels (Figure 4, Panel d). This suggests that the transfected cells are producing PIPs that are spreading through the culture to infect remaining naive cells. We next tested if we could use PIPs to transfer the replicons to mosquito C6/36 cells. Supernatant from BHK cells transfected with pBG78 and pBG44 were collected, clarified by centrifugation, and added to C6/36 cell cultures. The infected C6/36 cells were treated with blasticidin two days after infection, and were cultured from an additional 3 weeks in the presence of drug. GFP fluorescence was detected in adjacent cells 3 days after infection, and large colonies of GFP expressing cells were seen 3 weeks after infection (Figure 4, panels E and H). The number of GFP positive cells from the initial infection was relatively low following media transfer, but the formation of blasticidin resistant, GFP expressing colonies indicates that the replicon RNA can be transferred to naive insect cells and establish persistent infection.

**Figure 5:**
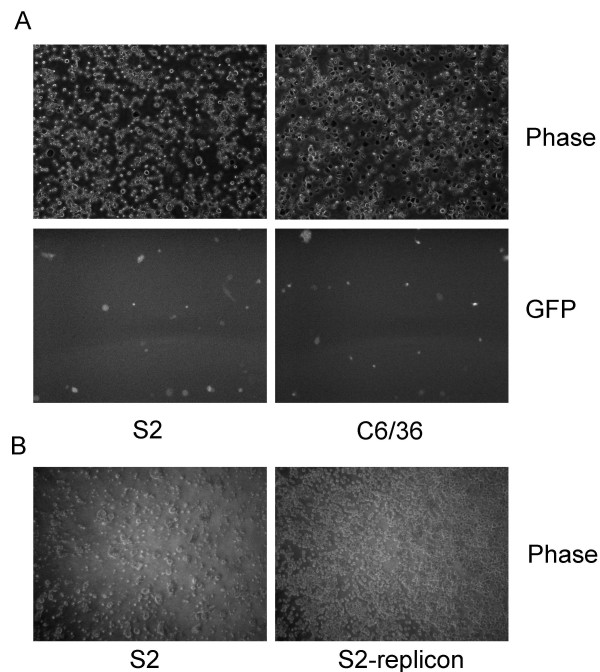
**Co-culturing S2 or C6/36 cells with PIP producing BHK cells increases infection efficiency**. 1 × 10^6 ^S2 or C6/36 cells were added to BHK cells co-transfected with pBG78 and pBG44 24 hrs post-transfection. Co-cultures were incubated for 24 hrs and the S2 or C6/36 cells were removed by gentle washing and transferred to new plates. GFP fluorescence was detected 24 hrs later (Panels A2 and A4) and 10 ug/ml added. **B**. Cells were incubated for 5 days in the presence of blasticidin and phase contrast micrographs obtained to assess cell viability.

To increase the efficiency of replicon transfer from BHK cells to insect cells, we established a BHK-target cell co-culture protocol using either C6/36 cells or *Drosophila melanogaster *S2 cells. This protocol takes advantage of the difference in adherence of mammalian and insect cells to plastic culture plates, allowing cells insect cells to be transiently co-cultured with BHK cells producing PIPs. BHK cells were transfected with pBG78 and pBG44 as described above. 24 hrs later the transfected BHK cells were washed with complete L-15 or Schneider's media and 1 × 10^6 m ^and C6/36 or Drosophila S2 cells were added directly onto the BHK cells. The co-cultures were incubated at 28°C for 24 hrs, and the C6/36 or S2 cells were separated from the BHK cells by gentle washing and were transferred to new plates. 24 hrs post-transfer 10 ug/ml blasticidin was added to the wells and GFP fluorescence detected. The number of GFP positive C6/36 cells was greatly enhanced as compared to C6/36 cells incubated with media from PIP producing BHK cells. We also observed a significant number of GFP positive S2 cells (Figure 5A). Seven days after blasticidin treatment no viable cells remained in a culture of co-culture mock infected S2, whereas there was no observed cell death in replicon co-culture infected S2 cells (Figure 5B). Similar data were observed for C6/36 cells (data not shown). Therefore, the replicons are transferred from the BHK cells to insect cells, and the co-culture assay is able to rapidly establish stable cell lines.

**Figure 6:**
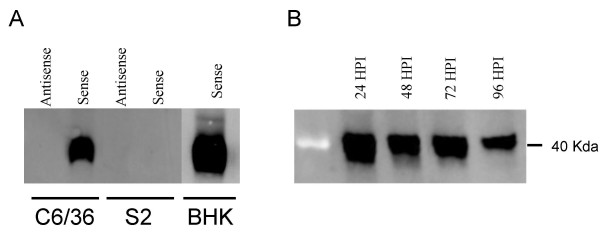
**SINV replicons can express epitope tagged mosquito genes for several days**. **A**. C6/36, S2, or BHK cells were infected with PIPs expressing a V5 epitope-tagged *Ae. aegypti *R2D2 cDNA either in a sense (pBG156) or antisense (pBG155) orientation. Cells were treated with blasticidin for four days and V5-R2D2 detected by western blot analysis. **B**. C6/36 cells were co-cultured with pBG156/pBG44 co-transfected BHK cells, transferred to new plates, and samples of cells collected at the indicated times for western blot analysis.

We used our co-culture protocol to express an epitope-tagged version of an endogenous *Aedes aegypti *RNAi component, R2D2. C6/36 or S2 cells were co-culture infected with PIPs produced from the R2D2 expressing replicon plasmid pBG156 or pBG155 (R2D2 antisense), and selected with blasticidin. Four days after blasticidin treatment 1 × 10^5 ^cells were collected, lysed, and the protein resolved on SDS-PAGE. Western blot analysis of the V5 epitope-tagged R2D2 protein showed high levels of expression in C6/36 cells (Figure 6A, lane 2). However, we were unable to detect any *Ae. aegypti *R2D2 protein in infected S2 cells (Figure 6A, lane 4). *Drosophila *R2D2 has been previously shown to be unstable in the absence of DCR2 [19], so these results may represent the inability of *Drosophila *DCR2 to stabilize *Ae. aegypti *R2D2. Interestingly, R2D2 is stable in the BHK cells used in for the co-culture experiments (Figure 6A, lane 5). We then tested if replicon-expressed R2D2 can be stably expressed in C6/36 cells for multiple days following infection. We were able to detect R2D2 expression for at least 4 days post co-culture without blasticidin treatment (Figure 6B), indicating either that the protein is very stable in C6/36 cells or that the replicon can express the protein for several days post-infection. These experiments indicate that double-subgenomic replicons can be used to express and study the biology of individual genes in insect cells in a rapid manner.

### Insert regions from persistently replicating replicons can be rapidly isolated and recombined into entry plasmids

**Figure 7:**
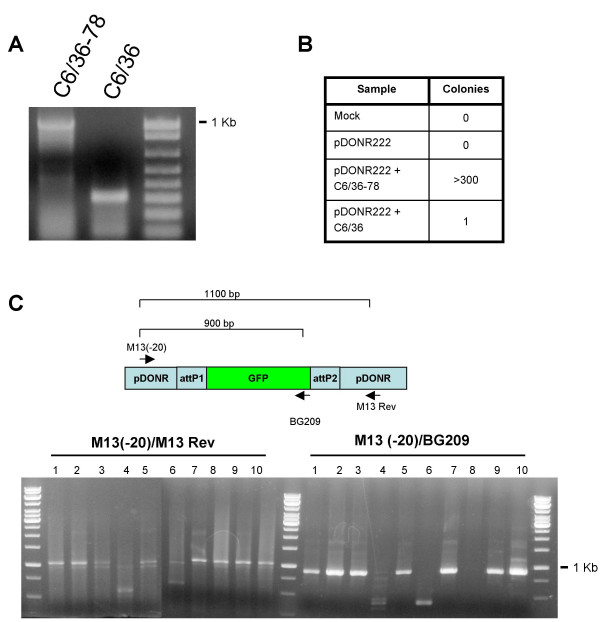
**Isolation of insert from replicon RNA**. **A**. RT-PCR product from C6/36–78 cells or naive C6/36 cells using primers BG162 and BG192. A non-specific amplification product was observed in C6/36 cells but not in C6/36–78 cells. **B**. Results of RT-PCR product recombination reactions transformed into *E. coli*. RT-PCR isolated cDNA was recombined into the pDONR222 entry plasmid and transformed into XL1-blue bacteria. **C**. Colony PCR from random isolated colonies from Figure 7B. The M13(-20)/M13 Rev primer set indicates the total insert size, and the M13(-20)/BG209 primer set detects the orientation of the GFP gene insert in the isolated plasmid.

We next wanted to determine if recombined inserts can be rapidly and efficiently recovered from persistently replicating replicons. The recombination reaction between the attL containing entry clone and the attR containing replicon expression plasmid results in the reconstitution of attB recombination sites competent for recombination with the attP sites in the pDONR222 plasmid. We isolated the attB containing GFP insert region from total RNA extracted from C6/36–78 cells in a RT-PCR reaction using replicon-specific primers (BG162 and BG192). A band of the appropriate size was present in the C6/36–78 cells and not in the C3/36 cells (Fig 7A). A nonspecific PCR product was generated from wildtype C6/36 cells that was not present in the C6/36–78 cells. The PCR products were purified using a PCR purification kit (Qiagen) and incubated with pDONR222 in a BP Clonase II reaction. The reaction product was transformed into DH5α *E. coli *cells and grown on LB + kanamycin plates. Figures 7B and 7C show the results of the transformation and the subsequent analysis of the clones. The reaction was very specific, as the recombination reaction with the non-replicon containing C6/36 cells resulted in very few colonies, whereas the recombination reaction with C6/36–78 derived RNA resulted in a large number of colonies. The majority of clones from the C6/36–78 recombination we tested contained the GFP gene in the expected orientation and the expected size (Figure 7C). Sequencing confirmed the presence of attB sites flanking the isolated GFP gene and the orientation of the gene (data not shown). Therefore the insert region from persistent replicons can be quickly and easily recovered from replicon-containing cells.

## Discussion

In this report we describe the construction and testing of a new recombination-based system for manipulating expression cassettes in Sindbis virus replicons and show their utility for expressing transgenes of interest in cultured mosquito cells. The replicon expressing plasmids we have developed have several features that make them unique, including replicon-expressed blasticidin drug resistance and the ability to specifically and efficiently insert a transgene of interest 3' to the second SGP by directional recombination.

The inclusion of the recombination cassettes into replicon expression plasmids make Sindbis replicons much easier to use compared to previously generations replicon expression plasmids. Because exogenous genes or DNA sequences can be inserted into the replicon by recombination rather than using restriction sites, it is much more likely that full-length genes can be inserted into the replicon. Such a feature becomes important if the replicon is to be used to express full-length genes, because large cDNAs are more likely to contain restriction sites that would make cloning the desired insert into the replicon more difficult. Additionally, recombination is directional and efficient. Almost all of the LR recombined replicon plasmids we tested contained the desired insert in the correct orientation. Finally, recombination is rapid. Once the DNA of interest has been recombined into an Entry plasmid, recombination of the attL flanked DNA into the attR containing replicons takes only 1 hour, in contrast to the many hours necessary for generating a replicon by restriction digestion and ligation.

There are several potential applications for recombination-compatible replicon plasmids. If an attL flanked cDNA library is generated from a cell or tissue of interest, that library can be recombined into the replicon plasmid in specifically in the sense orientation. A replicon expression library may be useful for expression clone screening that looks for genes introducing a new phenotype into a cell line, such as producing resistance to viral infection in a previously susceptible cell line. BP and LR recombination reactions appear to be scalable, so complex cDNA expression libraries can be generated with all of the inserts in the correct orientation for expression. The inclusion of a mutation at nsP2 amino acid 726 makes the replicon noncytopathic in mammalian cells ([20] and data not shown), which can allow a replicon cDNA expression library to be used in mammalian cells, although doing so appears to reduce the replication rate of the replicon. Coupling expression library replicons with an easily identified phenotypic readout, such as phenotype-induced GFP expression or altered cell viability, can be used to rapidly identify and enrich for cells with replicons expressing proteins affecting the phenotype. Once the cells of interest are enriched or isolated, the antisense-expression cassettes can be isolated from replicon RNA by reverse transcription, recombined into new entry plasmids, and either sequenced or recombined into new replicon plasmids for further rounds of screening. Because the replicons can incorporate and maintain fairly large inserts in plasmid form, it is possible that full-length genes can be directly isolated from antisense screening and immediately used for further testing. The percentage of cells that are infectable using our co-culture protocol is relatively high, increasing their utility in screening protocols.

Another interesting possibility is to use an antisense-expressing replicon library to screen for genes involved in particular cellular processes such as metabolism or viral infectability. Because replicons persistently replicate in cells, the expression of antisense RNA in selected replicon cells in theory could result in a "knock-out" phenotype. We have previously shown that expression of antisense RNA from a portion of the dengue virus genome drastically reduces the ability of cells and mosquitoes to be infected by dengue virus [2] and SINV mediated expression of antisense RNA to the Broad-Complex transcription factor in silkmoths was used to demonstrate the critical role of the protein in insect morphogenesis [21], and to assess the role of prophenoloxidase in mosquito melanization [22,23]. SINV antisense expression is therefore a valuable tool for the functional evaluation of gene function in insects. Further evaluation of the utility of antisense expressing SINV replicons needs to be performed to determine their utility in reducing gene expression in insect cells.

The recombination-ready replicon expression plasmids detailed in this report provide convenient and efficient tools to the use of alphavirus replicons to address many biological questions in insect cells.

## Abbreviations

Sindbis virus (SINV); pseudo-infectious particles (PIPs); subgenomic promoter (SGP); hepatitis delta virus (HDV); Green fluorescence protein (GFP); cytomegalovirus immediate early promoter (CMV)

## Competing interests

The author(s) declare that they have no competing interests.

## Authors' contributions

LHS contributed to the development of the pBG60 plasmid and provided critical evaluation of the manuscript. CIS performed experiments with *Ae. Aegypti *R2D2 gene and provided critical evaluation. BJG designed the study, performed many of the experiments in the manuscript, and drafted the manuscript. KEO assisted in the design of the study, acquired funding for the project, and provided critical analysis of the manuscript. All authors have read and approved the final manuscript.
